# Marginalizing the genomic architecture to identify crosstalk across cancer and neurodegeneration

**DOI:** 10.3389/fnmol.2023.1155177

**Published:** 2023-02-27

**Authors:** Amit Sharma, Ullrich Wüllner, Ingo G. H. Schmidt-Wolf, Jarek Maciaczyk

**Affiliations:** ^1^Department of Neurosurgery, University Hospital of Bonn, Bonn, Germany; ^2^Department of Neurology, University Hospital of Bonn, German Center for Neurodegenerative Diseases (DZNE), Bonn, Germany; ^3^Department of Integrated Oncology, Center for Integrated Oncology (CIO), University Hospital of Bonn, Bonn, Germany; ^4^Department of Surgical Sciences, Dunedin School of Medicine, University of Otago, Dunedin, New Zealand

**Keywords:** cancer, neurodegeneration, genome, epigenome, pathway

Cancer and neurodegenerative diseases (NDD) appear mechanistically distinct, i.e., the former acquires mechanisms to resist and evade cell death, while the latter is characterized by progressive cellular demise and degeneration in specific neuronal populations. Nevertheless, there is an ongoing debate about the inverse epidemiologic relationship between cancer and NDD. A substantial number of cancer survivors have a lower risk of developing NDD, particularly Parkinson's disease (PD) and Alzheimer's disease (AD), whereas less malignancies are observed in PD and AD patients. Several biological hypotheses have been put forward (Wang et al., 2013; Catalá-López et al., 2014; Majd et al., 2019; Panegyres and Chen, 2021; Zabłocka et al., 2021; Lee et al., 2022), but exact underlying mechanisms behind this “*inverse association*” remain to be explored. Interestingly the correlation between cancer and AD appears to be purely negative/inverse, suggesting that susceptibility to one disease may be protective against the other (Musicco et al., 2013; Driver, 2014; Zhang et al., 2015; Papageorgakopoulos et al., 2017). Contrary in PD, patients showed a low risk to develop colon, rectal, colorectal cancer and lung cancers but increased risks of brain cancer and melanoma (Ye et al., 2020). The possible involvement of certain genes and signaling pathways behind this inverse comorbidity has been discussed (Ibáñez et al., 2014). In particular, the authors elaborated on the possible roles of Wnt and p53 signaling pathways and protein degradation processes (Ubiquitin/proteasome system) underlying the observed differences in CNS diseases and cancers. The putative role of non-coding genomes (LnRNAs, miRNAs) has also been briefly investigated. Pandini et al. (2021), recently discussed the mechanisms of action associated with MYC-induced long non-coding RNA (MINCR) and its potential implications in both cancer and NDD. Likewise, miR-519a-3p, which is normally upregulated in certain cancers, appears to be downregulated in PD (*via* possible engagement of its target gene PARP1) (Salemi et al., 2022).

Since most NDD and cancer patients are sporadic, the notion of inverse association in familial cases is still unexplored. Notably, mutations in the gene encoding leucine-rich repeat kinase 2 (LRRK2) are associated with both familial and sporadic PD. There is plethora of knowledge indicating the “*overlapping*” molecular mechanisms between these two entities. For instance, TP53—the most frequently mutated gene in human cancers, often named as—“guardian of the genome” (Chen et al., 2022), turns out to be one of the discriminating tools also in NDD (Chang et al., 2012; Checler and da Alves Costa, 2014; Talebi et al., 2021). It has been shown that the mononuclear cells from AD patients express higher levels of mutant-like p53 (conformationally altered p53) compared to non-AD patients (Lanni et al., 2008). Likewise, p53 protein levels were found markedly elevated in caudate nucleus of PD patients (Mogi et al., 2007). Similar to TP53 PIN1 (Peptidyl-prolyl isomerase), has been reported to inactivate and activate more than 26 tumor suppressors and 56 oncogenes, in numerous malignancies (Yu et al., 2020). Besides cancer, a number of studies highlighted the possible involvement of PIN1 in NDDs (Pastorino et al., 2006; Ryo et al., 2006). Driver et al. (2015) discussed the diverse priorities of PIN1 in cyclic cells and neurons, and suggested that understanding its role may explain the inverse association between cancer and AD. Of interest, in cancers that originate mainly in the brain (e.g., glioblastoma/GBM), an obvious communication between the cancer cells and adjacent neuronal cells can be expected. In such scenario, some genetic/molecular resemblances shared by both cancer and neuronal cells would not be surprising, and genes such as PARK7 (which encodes the protein DJ-1) fit well into this scenario. More specifically, whereas the mutation/deletion of PARK7 leads to the early onset of PD (Dolgacheva et al., 2019), this gene seems to play a role in cancerogenesis (Kawate et al., 2017). Particularly in GBM, the immunohistochemical staining showed enhanced expression of PARK7 in glioma tissues compared to the normal brain tissues (Kim et al., 2021). Thus, a multifunctional protein like PARK7 represents a prime candidate explaining the pathological interactions between cancer (GBM) and PD, which occur in the anatomically different regions yet in same organ. Similarly, Tau protein (encoded by the MAPT gene), one of the major hallmarks of AD, is assumed to bind cancer-related kinase proteins due to its ability to accumulate both intracellularly and extracellularly (Papin and Paganetti, 2020; Hedna et al., 2022). Certain miRNAs are also instrumental in these overlapping mechanisms. Notably, miRNAs that are differentially expressed in NDDs (e.g., miR-9, the miR-29 family, and the miR-34 family) have also been implicated as potential tumor suppressors (Saito and Saito, 2012). Strikingly, the substantial epigenetic constraint on cancer progression appears to be a mediating rather than a causative factor when compared with NDD. For instance, rapid divisions in cancer cells requires a continuous rewriting of epigenetic marks (e.g., DNMTs, HAT/HDACs) in their daughter cells, whereas in NDD all the established epigenetic marks vanished with the loss/degeneration of neuronal cells.

Beyond the aforementioned—*inverse* or *overlapping*—mechanisms, another avenue that remains to be explored is the identification of distant molecular contributors involved in these two entities. A prerequisite for such possible causative contributors should be their ability to play a dichotomous functional role in cancer (i.e., both tumor suppressor and tumor promoter), having open access to the epigenetic landscape/chromatin machinery (to control transcriptional activity) and a strong tendency to cross-talk with other non-cancerous hallmarks. Interesting candidate are the ubiquitin C-terminal hydrolases (UCHs: UCH-L1, UCH-L3, UCH-L5, and BAP1), a subfamily of deubiquitinating enzymes, which we have recently shown to be involved in both cancer and NDD (Sharma et al., 2020). Specifically, UCH-L1 and BAP1 are of interest. UCH-L1 (also known as PARK5 and PGP9.5) was previously found to be enriched in neurons, shown to promote alpha-synuclein neurotoxicity in PD patients (Liu et al., 2009) at the same time being proposed as an oncogene (Hurst-Kennedy et al., 2012; Zhong et al., 2012). Likewise, BAP1 gene has been implicated in several types of cancer and is considered pivotal to constrain histone H2A monoubiquitylation (H2AK119ub1) in the genome (Sharma et al., 2019; Fursova et al., 2021). Several cancer-associated mutations within this gene were found to destabilize the protein structure and promoted β-amyloid aggregation *in vitro*, constituting a pathological hallmark of AD (Bhattacharya et al., 2015). Though apparently distant (not directly linked), understanding the multifaceted involvement of UCH-L1 and BAP1 in cancer and NDD could be of importance. Other potential candidates are adenosine receptors (ARs), a family of G protein-coupled receptors (GPCRs) whose four (A1, A2A, A2B, and A3) members have all been involved in one way or another in the regulation of tumor progression (Franco et al., 2021). A1 and A2A show the highest expression in the brain but their relevance for NDD has yet to be defined (Stockwell et al., 2017).

It is widely accepted that NDD start long before clinical symptoms (Sharma et al., 2021), i.e., parkinsonism or dementia arise and affect the patients substantially, thus tracking the subsequent overt disease progression or severity may not reveal information relevant to the primary cause (Surguchov, 2022). Similarly, in cancer it appeared crucial to determine the “*disease onset point*” by understanding the aforementioned overlapping/inverse molecular mechanisms. Given the rapid development of sequencing technology it can be envisioned that defining genotype of sporadic and familial cases across populations may improve insight into the shared genetic architecture connecting disease specific phenotypes, ranging from cancer to NDD. Consequently, such approaches will also help identify aging-related exponential accumulation of mutations that may possibly be correlated with the proteins/proteolytic fragments released by degenerating neurons in order to develop advanced novel therapies.

## Author contributions

All authors listed have made a substantial, direct, and intellectual contribution to the work and approved it for publication.
